# Socioeconomic, meteorological factors and spatiotemporal distribution of human brucellosis in China between 2004 and 2019—A study based on spatial panel model

**DOI:** 10.1371/journal.pntd.0011765

**Published:** 2023-11-13

**Authors:** Zi-Xin Sun, Yan Wang, Ying-Jie Li, Shi-Hao Yu, Wei Wu, De-Sheng Huang, Peng Guan

**Affiliations:** 1 Key Laboratory of Environmental Stress and Chronic Disease Control & Prevention (China Medical University), Ministry of Education, Shenyang, China; 2 Department of Epidemiology, School of Public Health, China Medical University, Shenyang, China; 3 Department of Intelligent Computing, School of Intelligent Medicine, China Medical University, Shenyang, China; Colorado State University, UNITED STATES

## Abstract

**Background:**

Human brucellosis continues to be a great threat to human health in China. The present study aimed to investigate the spatiotemporal distribution of human brucellosis in China from 2004 to 2019, to analyze the socioeconomic factors, meteorological factors and seasonal effect affecting human brucellosis incidence in different geographical regions with the help of spatial panel model, and to provide a scientific basis for local health authorities to improve the prevention of human brucellosis.

**Methods:**

The monthly reported number and incidence of human brucellosis in China from January 2004 to December 2019 were obtained from the Data Center for China Public Health Science. Monthly average air temperature and monthly average relative humidity of 31 provincial-level administrative units (22 provinces, 5 autonomous regions and 4 municipalities directly under the central government) in China from October 2003 to December 2019 were obtained from the National Meteorological Science Data Centre. The inventory of cattle, the inventory of sheep, beef yield, mutton yield, wool yield, milk yield and gross pastoral product of 31 provincial-level administrative units in China from 2004 to 2019 were obtained from the National Bureau of Statistics of China. The temporal and geographical distribution of human brucellosis was displayed with Microsoft Excel and ArcMap software. The spatial autocorrelation and hotspot analysis was used to describe the association among different areas. Spatial panel model was constructed to explore the combined effects on the incidence of human brucellosis in China.

**Results:**

A total of 569,016 cases of human brucellosis were reported in the 31 provincial-level administrative units in China from January 2004 to December 2019. Human brucellosis cases were concentrated between March and July, with a peak in May, showing a clear seasonal increase. The incidence of human brucellosis in China from 2004 to 2019 showed significant spatial correlations, and hotspot analysis indicated that the high incidence of human brucellosis was mainly in the northern China, particularly in Inner Mongolia, Shanxi, and Heilongjiang. The results from spatial panel model suggested that the inventory of cattle, the inventory of sheep, beef yield, mutton yield, wool yield, milk yield, gross pastoral product, average air temperature (the same month, 2-month lagged and 3-month lagged), average relative humidity (the same month) and season variability were significantly associated with human brucellosis incidence in China.

**Conclusions:**

The epidemic area of human brucellosis in China has been expanding and the spatial clustering has been observed. Inner Mongolia and adjacent provinces or autonomous regions are the high-risk areas of human brucellosis. The inventory of cattle and sheep, beef yield, mutton yield, wool yield, milk yield, gross pastoral product, average air temperature, average relative humidity and season variability played a significant role in the progression of human brucellosis. The present study strengthens the understanding of the relationship between socioeconomic, meteorological factors and the spatial heterogeneity of human brucellosis in China, through which ‘One Health’-based strategies and countermeasures can be provided for the government to tackle the brucellosis menace.

## Author summary

Human brucellosis continues to be a great threat to human health in China. It was reported that human brucellosis incidence in China was 4.95/100,000 in 2021, surging from 16^th^ place in 2000 to 5^th^ place in 2021. The present study was designed to investigate the spatiotemporal distribution of human brucellosis in China from 2004 to 2019, to analyze the socioeconomic factors, meteorological factors, and seasonal effect affecting human brucellosis incidence in different geographical regions. The clear seasonal increase of reported human brucellosis cases between March and July, the spatial clustering situation and the spread of epidemic area from northern to the southern coast and southwestern regions were identified. The inventory of cattle and sheep, beef yield, mutton yield, wool yield, milk yield, gross pastoral product, average air temperature (the same month, 2-month lagged and 3-month lagged), average relative humidity (the same month) and season variability played a significant role in the progression of human brucellosis. Spatial panel model was found to have good applicability and interpretability. As each factor plays a different role in different geographical regions, a comprehensive surveillance system for human brucellosis should be established in conjunction with the relevant risk factors to provide early warning of human and animal epidemics.

## Introduction

Brucellosis is a zoonosis caused by *Brucella* and is classified as one of the category B legally reported infectious diseases in China [1]. The main sources of infection for animal brucellosis are sheep, cattle, pigs and other animals infected with *Brucella*. Direct contact or consumption of infected animals and their products are the main routes of infection for humans [2–4]. In general, all human beings are generally susceptible to *Brucella*. Although animal brucellosis has been declared eradicated in developed countries such as Japan, Australia and Canada, it is still highly prevalent in the Middle East, the Mediterranean coast, Asia, most of the Africa and Latin America [5]. As the economies of these countries were heavily dependent on animal husbandry, and due to the large number of livestock and the wide spread of livestock products, the economy suffered a great loss caused by brucellosis [6]. For China’s herbivorous animal husbandry industry, the clear trend of moving from pastoral area to agricultural area has been identified over the past four decades [7]. According to the results of an epidemiological survey of 448,398 domestic animals in Qingyang, China, it has reported that the animal brucellosis prevalence ranged from 0 to 9.96%, with sheep being the primary host of *Brucella* [8]. Since the 1990s, human brucellosis has made a comeback in China, the incidence of human brucellosis in China was 4.95/100,000 in 2021, surging from 16^th^ place in 2000 to 5^th^ place in 2021, and the trend was rare among the notifiable infectious diseases [9,10]. The high incidence of human brucellosis was mainly concentrated in the northern regions, however, the epidemic area has tended to spread from the northern pastoral areas to the adjacent grassland and agricultural areas, so that the affected areas in the south have gradually increased in recent years [11]. The serious situation drives the need to continue to study the relevant driving factors of human brucellosis.

There have been a series of studies on the driving factors of human brucellosis. For instance, Cao *et al*. [12] reported that human brucellosis incidence was positively correlated with wind speed and negatively correlated with air pressure and average air temperature. Peng *et al*. [13] reported that human brucellosis incidence was significantly correlated with an increase in average air temperature and normalized difference vegetation index (NDVI), and a decrease in mean precipitation. However, Liang *et al*. [14] found that human brucellosis incidence was associated with a larger number of cattle and sheep, lower gross domestic product (GDP), and fewer hospital beds, the correlation between human brucellosis incidence and relative humidity, average precipitation, and average sunshine hours was not statistically significant. It can be seen that these socioeconomic factors and meteorological factors played different roles in different regions and the results varied widely. Most of the previous studies focused either on spatial distribution or on temporal analysis, which to some extent led to the lack of information and inconsistent conclusions. Thus, it is of great importance to take account of both spatial and temporal effect when exploring the driving factors of the diseases. Furthermore, adjacent regions often share similar geographical, economic and climatic characteristics, also there may be more frequent animal population movements or livestock trading activities, so cross-regional joint cooperation on brucellosis control helps enhance the early warning and increase the control efficiency [15]. The aim of this study was thus to investigate the spatiotemporal distribution of human brucellosis in China, to adopt an ecological study design to analyze the socioeconomic factors, meteorological factors and seasonal effect affecting human brucellosis incidence in different geographical regions with the help of spatial panel model, and to provide a scientific basis for local health authorities to improve disease prevention measures.

## Methods

### Ethics statement

Research Institutional Review Board of China Medical University approved the research protocol and determined that the collection of all the raw data from publicly available sources was exempt from institutional review board assessment. The approved ethics protocol number was NSFC-71974199. All the data that were collected and analyzed in the present study did not involve any personal identifying information.

### Human brucellosis and geographical data

**Fig 1:**
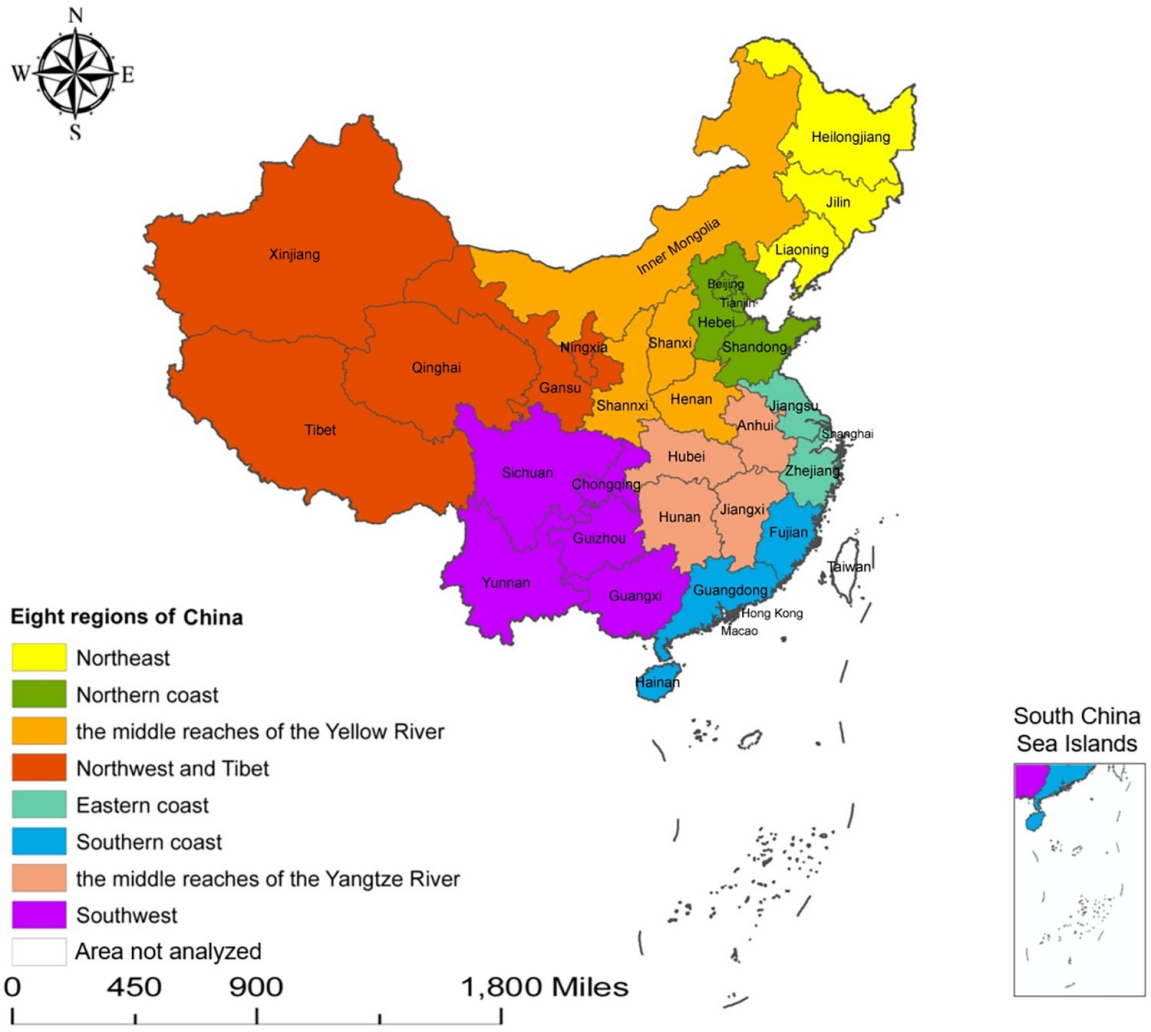
Eight geographical regions of China for the analysis of human brucellosis incidence in the spatial panel model. Base layers were downloaded from Standard Map Service System by China Cartographic Publishing House (http://bzdt.ch.mnr.gov.cn/). No. GS(2020)4619. Note: the map of this study does not represent the true borders of administrative regions of China.

The monthly number and incidence of human brucellosis in 31 provincial-level administrative units (22 provinces, 5 autonomous regions, 4 municipalities directly under the central government) in China from January 2004 to December 2019 were obtained from the Data Center for China Public Health Science (https://www.phsciencedata.cn/Share/). Thirty-one provincial-level administrative units, monthly data for 16 consecutive years, so a total of 5952 data entered the current ecological study. The map of China was downloaded from Standard Map Service System by China Cartographic Publishing House (http://bzdt.ch.mnr.gov.cn/). As China is a climatologically and economically diverse country, according to the previous study, we divided the 31 provincial-level administrative units in China into eight geographical areas, including Northeast, Northern coast, the middle reaches of the Yellow River, Northwest and Tibet, Eastern coast, Southern coast, the middle reaches of the Yangtze River, and Southwest [16] (see Fig 1). Among the eight regions, when analyzed in terms of two parts: the North and the South, Northeast, Northern coast, the middle reaches of the Yellow River and Northwest and Tibet belonged to North of China, while Eastern coast, Southern coast, the middle reaches of the Yangtze River and Southwest were considered as South of China [17].

### Socioeconomic and meteorological data

The inventory of cattle, the inventory of sheep, beef yield, mutton yield, wool yield, milk yield and gross pastoral product of 31 provincial-level administrative units in China from 2004 to 2019 (Table 1) were considered in the study and obtained from the National Bureau of Statistics of China (https://data.stats.gov.cn/). Monthly average air temperature (°C) and average relative humidity (%) from January 2004 to December 2019 were obtained from the National Meteorological Science Data Centre (http://data.cma.cn/). Based on the previous study, the maximum length of lag period was set to three months [18]. Thus, in order to examine the lagged effects of meteorological factors, monthly average air temperature and average relative humidity from October to December in 2003 were also collected in this study (S1 Table).

**Table 1:** Descriptions of yearly socioeconomic factors in China from 2004 to 2019.

Geographical regions (Number of provincial-level administrative units in each region)	Population [million, median (range)]	Inventory of cattle [million heads, median (range)]	Inventory of sheep [million heads, median (range)]	Beef yield [million tons, median (range)]	Mutton yield [million tons, median (range)]	Wool yield [thousand tons, median (range)]	Milk yield [million tons, median (range)]	Gross pastoral product [billion Chinese Yuan, median (range)]
North								
Northeast (3)	108.908 (107.430, 109.763)	13.198 (10.301, 15.130)	13.326 (10.636, 19.532)	1.214 (1.070, 1.312)	0.238 (0.191, 0.263)	56.535 (37.652, 66.689)	6.720 (4.583, 7.631)	355.335 (134.802, 439.091)
Northern coast (4)	203.530 (185.060, 213.780)	9.502 (7.481, 18.670)	16.922 (12.324, 30.884)	1.312 (1.241, 1.782)	0.670 (0.564, 0.771)	43.857 (26.099, 60.010)	8.247 (5.516, 8.958)	419.520 (203.335, 470.820)
the middle reaches of the Yellow River (4)	192.325 (188.281, 197.850)	18.393 (12.415, 26.694)	47.668 (35.064, 95.887)	1.440 (1.088, 1.532)	1.271 (1.098, 1.552)	128.529 (110.770, 152.634)	13.333 (7.297, 14.480)	405.504 (181.253, 494.277)
Northwest and Tibet (5)	63.111 (59.075, 68.240)	19.884 (19.011, 21.950)	74.987 (63.576, 90.533)	0.838 (0.658, 1.145)	0.965 (0.835, 1.154)	144.536 (126.811, 170.064)	3.295 (2.479, 5.091)	94.019 (35.187, 186.788)
South								
Eastern coast (3)	157.433 (142.832, 163.480)	0.585 (0.461, 1.063)	0.828 (0.596, 4.867)	0.044 (0.039, 0.072)	0.099 (0.090, 0.224)	2.357 (1.839, 2.731)	1.047 (0.966, 1.108)	153.094 (86.978, 186.825)
Southern coast (3)	151.654 (134.578, 164.390)	3.901 (2.015, 6.499)	0.0 (0.0, 2.669)	0.110 (0.077, 0.131)	0.040 (0.030, 0.054)	-	0.289 (0.275, 0.318)	193.699 (94.665, 261.937)
the middle reaches of the Yangtze River (4)	229.089 (224.670, 238.770)	12.013 (9.394, 18.254)	0.028 (0.008, 19.239)	0.637 (0.529, 0.777)	0.346 (0.285, 0.469)	0.159 (0.134, 0.239)	0.574 (0.401, 0.731)	430.674 (198.022, 604.242)
Southwest (5)	239.964 (236.201, 249.400)	27.836 (25.338, 36.484)	3.323 (2.688, 37.413)	0.938 (0.732, 1.166)	0.470 (0.378, 0.623)	8.370 (7.401, 10.844)	1.409 (0.964, 1.501)	500.522 (226.346, 694.739)

Note: ‘-’ means no data of wool yield in that region.

### Descriptive analysis

In order to characterize the temporal distribution and seasonal characteristics of human brucellosis in China, we used Microsoft Excel 2021 software to present the reported incident cases of human brucellosis from January 2004 to December 2019, and ArcMap 10.7 software was used to create a map to show its geographical distribution.

### Spatial autocorrelation and hotspot analysis

Spatial autocorrelation analysis can describe the association among different areas and has gradually become an important method for the characterization of infectious disease clustering, of which the Moran index (Moran’s *I*) is one of the classical indicators for spatial autocorrelation [19,20] The global spatial autocorrelation (global Moran’s *I*) was used to assess the overall autocorrelation of different regions in China, and the local spatial autocorrelation (local Moran’s *I* and Getis-Ord Gi* statistics) was used to analyze the correlation between a region and its surrounding areas, and thus to obtain the local clustering of human brucellosis in that region. The Getis-Ord-Gi* statistic is a spatial autocorrelation index for hotspot analysis based on a weighted distance matrix that uses high (hot spots) or low (cold spots) values to determine the spatial clustering of locations [21].

The global Moran’s *I*, local Moran’s *I* and Getis-Ord-Gi* statistic can be calculated according to the following formula respectively:

$$I=\frac{n}{\sum_{i=1}^{n}\sum_{j=1}^{n}w_{ij}}\times \frac{\sum_{i=1}^{n}\sum_{j=1}^{n}w_{ij}{\tilde{y}}_{i}{\tilde{y}}_{j}}{{\sum_{i=1}^{n}{\tilde{y}}_{i}}^{2}}$$ (1)

$$I_{i}=\frac{{\tilde{y}}_{i}}{\frac{1}{n}{\sum_{i=1}^{n}{\tilde{y}}_{i}}^{2}}\times \sum_{j\neq i}^{n}w_{ij}{\tilde{y}}_{j}$$ (2)

$$G_{i}^{*}=\frac{\sum_{j=1}^{n}\;w_{ij}y_{j}-\bar{y}\sum_{j=1}^{n}\;w_{ij}}{\sqrt{\frac{\sum_{j=1}^{n}\;y_{j}^{2}}{n}-{\left(\bar{y}\right)}^{2}}\times \sqrt{\frac{n\sum_{j=1}^{n}\;w_{ij}^{2}-{\left(\sum_{j=1}^{n}\;w_{ij}\right)}^{2}}{n-1}}}$$ (3)

where *n* is the number of provincial-level administrative units analyzed in the present study in China, *y*_*i*_ denotes the human brucellosis incidence of the *i*th region, $\bar{y}$ is the mean value of the incidence of all geographical regions, *w*_*ij*_ is the spatial weight of the *i*th region and the *j*th region. Queen contiguity is used, where *w*_*ij*_ is defined as “1” for those which share the same boundary and vertices in each geographical region, and “0” otherwise [22]. *I*_*i*_ is the local Moran’s *I* of the *i*th region, and ${\tilde{y}}_{i}=y_{i}‐\bar{y}$ is the difference between the incidence of the *i*th region and $\bar{y}$.

The significance of the Moran’s *I* and Getis-Ord Gi* statistics is tested using the *z*-value with the significance level of 0.05 [23]. If *z*-value > 0, it refers to a positive spatial correlation. The higher the z-value, the tighter the clustering of high values (hot spots of human brucellosis incidence in the present study), and the lower the *z*-value, the tighter the clustering of low values (cold spots of human brucellosis incidence in the present study). There were four possible types of local clustering: High-High Cluster, Low-Low Cluster, Low-High Outlier and High-Low Outlier, where High-High Cluster was the high incidence of clustering, Low-Low Cluster was the low incidence of clustering, and Low-High and High-Low Outlier suggested the abnormal distribution. The results were visualized by local indications of spatial association (LISA) cluster map (S2 Fig). The above analyses were performed by ArcMap 10.7 software.

### Spatial panel model

Panel data can be regarded as a mixture of time series and cross-sectional data, which are repeated observations of different individuals in the cross-section at different points in time, and therefore it is two-dimensional data. Panel data models can effectively solve the problem of data heterogeneity and reveal the time-varying characteristics of variables, and have been widely used in economics, new drug testing and many other fields [24]. Spatial panel model is applicable to spatially dependent data and can deal with spatial and temporal heterogeneity. It controls for multicollinearity and takes a broader view of the problem [25]. A common spatial panel model can be represented by the following equation:

$$\left\{ \begin{array}{l} y_{it}=\rho W_{i}y_{t}+x_{it}\beta +d_{i}x_{t}\delta +u_{i}+\gamma_{t}+\varepsilon_{it}\\ \varepsilon_{it}=\lambda m_{i}\varepsilon_{t}+v_{it} \end{array}\right.$$ (4)

where *y*_*it*_ is the logarithm of incidence of human brucellosis in the *i*th region at time *t*, ***x***_*it*_ is the independent variable *x* in the *i*th region at time *t*, ***β*** is the regression coefficient, *ρ* denotes the spatial autoregressive coefficient, and *λ* is the spatial error. ***W***_*i*_ is an *n*×*n* spatial weight matrix, and weights are row-standardized. ***d***_*i*_ is the *i*th row of the spatial weight matrix for *y*, and ***m***_*i*_ is the *i*th row of the spatial weight matrix for disturbance. *u*_*i*_ represents the individual effect, *γ*_*t*_ represents the time effect, and *ε*_*it*_ is the random error. In the spatial weight matrix, Queen contiguity is used, and Hainan province is bordered by Guangdong province through the Qiongzhou Strait. Specifically, if *λ* = 0, it is called Spatial Durbin Model (SDM). If *λ* = 0 and ***δ*** = 0, it becomes Spatial Lag Model (SLM). If *ρ* = 0 and ***δ*** = 0, it becomes Spatial Error Model (SEM).

In the spatial panel models, human brucellosis incidence was used as the dependent variable, and the candidate independent variables with the smallest temporal unit of data clustering included the inventory of cattle (yearly), the inventory of sheep (yearly), beef yield (yearly), mutton yield (yearly), wool yield (yearly), milk yield (yearly), gross pastoral product (quarterly), the same month’s and one to three month(s) lagged average air temperature, the same month’s and one to three month(s) lagged average relative humidity and seasonal effect (S1, S2, S3). We set dumb variables S1, S2, S3 to represent spring (March–May), summer (June–August), autumn (September–November) respectively, with reference to winter (December–February).

In order to explore the ranking of the variables’ contribution, the dependent and independent variables were standardized using the following equation:

$$x^{\prime }=(x-\mu )/\sigma$$ (5)

where *x*′ is the value after standardization, *x* is the original value, *μ* is the average value of *x*, and *σ* is the standard deviation of *x*.

In order to control the effect of multi-collinearity among variables, multi-collinearity test was conducted to remove those variables with severe covariance. Variables with condition index >15 as well as variance ratio > 0.5 were removed from the model. Before constructing the spatial panel model, Lagrange multiplier (LM) and robust Lagrange multiplier (robust LM) test was used to determine whether SEM, SLM or SDM could be run. Panel data model can be classified as fixed effects model, random effects model and mixed effects model [26], which is determined by Hausman test [25]. A *p-*value < 0.05 was considered statistically significant. The above analyses were performed in R 4.2.1 software (*splm*, *spdep* package).

## Results

### Characteristics of temporal distribution of human brucellosis

**Fig 2:**
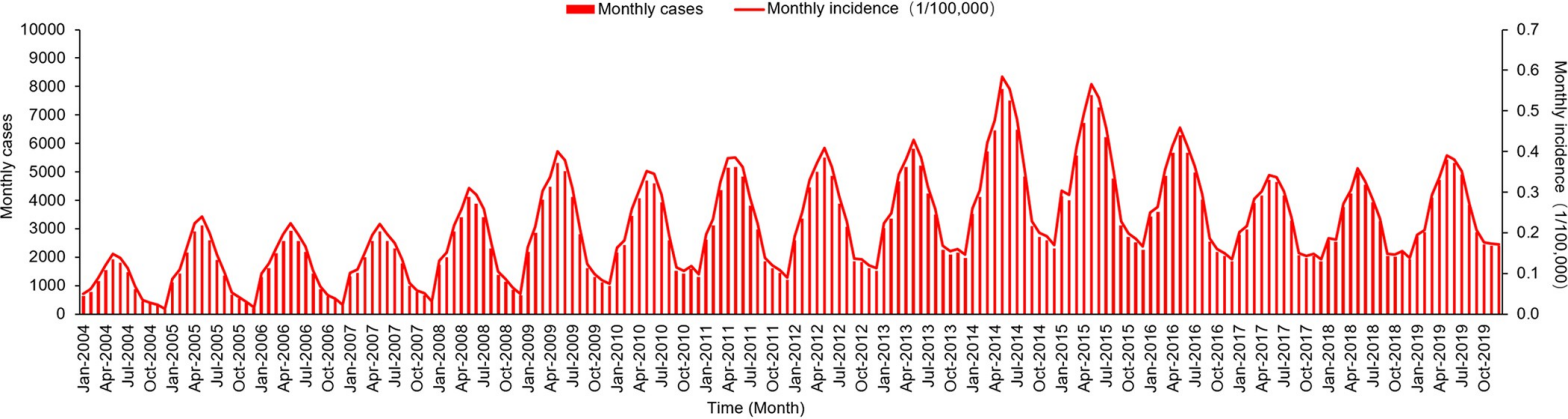
Temporal distribution of human brucellosis in China from 2004 to 2019.

**Fig 3:**
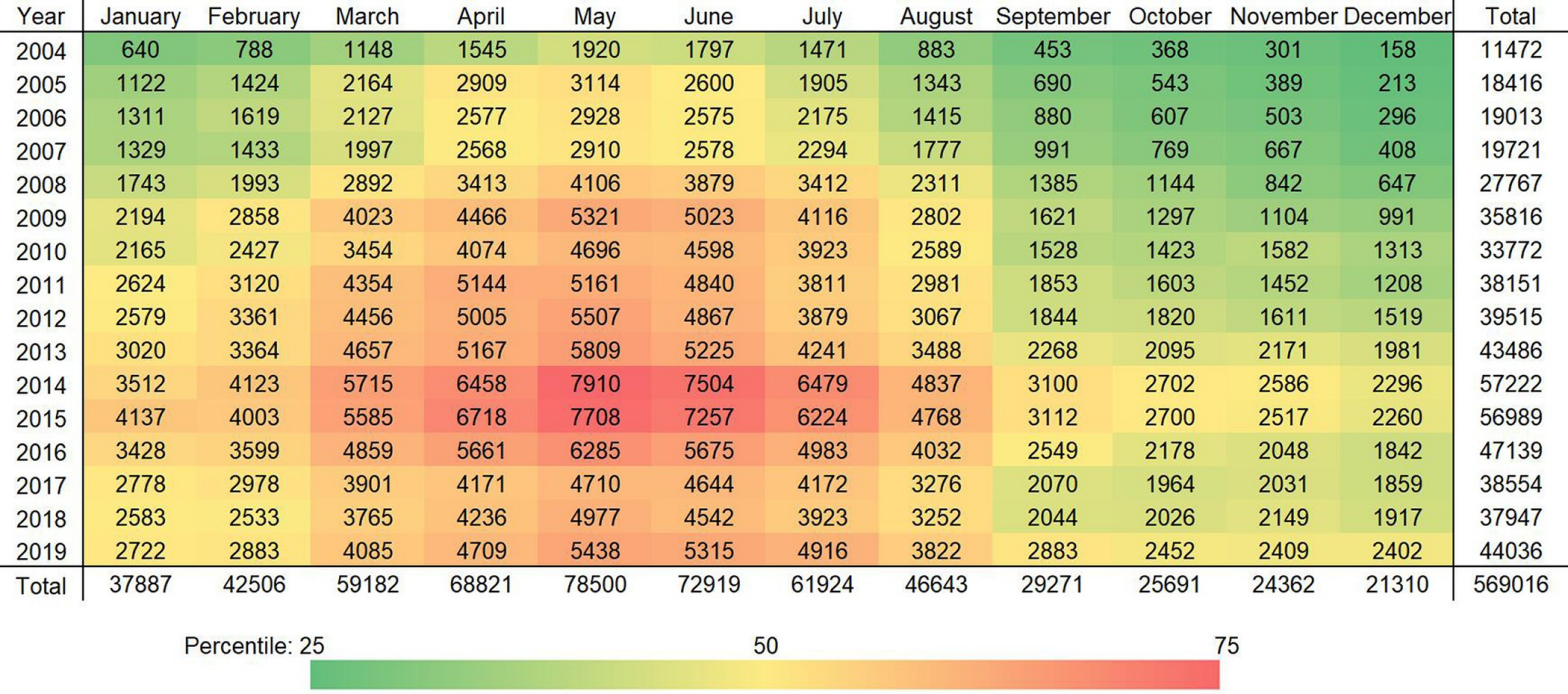
Heat map of monthly human brucellosis incident cases in China from 2004 to 2019.

A total of 569,016 cases of human brucellosis were reported in China from January 2004 to December 2019. The incidence of human brucellosis has shown an overall upward trend since 2004 (0.88/100,000), peaked in 2014 (4.22/100,000), and then declined to 2.73/100,000 in 2018 (Fig 2). Human brucellosis cases were concentrated between March and July, with a peak in May, showing a clear seasonal increase (Fig 3).

Human brucellosis was mainly concentrated in the northern parts of China before 2011, and rapidly spread to the southeastern coast and southwest of China from 2011, and had spread throughout China by 2013 (S1 Fig).

The top five provincial-level administrative units in terms of human brucellosis incidence from 2004 to 2019 were all located in the northern China, with the incidence in Inner Mongolia, Shanxi and Heilongjiang consistently ranked in this list during the 16-year period (Table 2).

**Table 2:** Top five provincial-level administrative units in China in terms of human brucellosis incidence from 2004 to 2019.

Year	Ranking				
	1^st^	2^nd^	3^rd^	4^th^	5^th^
2004	Inner Mongolia	Tibet	Heilongjiang	Shanxi	Liaoning
2005	Inner Mongolia	Heilongjiang	Shanxi	Jilin	Hebei
2006	Inner Mongolia	Shanxi	Heilongjiang	Hebei	Jilin
2007	Inner Mongolia	Shanxi	Heilongjiang	Hebei	Jilin
2008	Inner Mongolia	Shanxi	Heilongjiang	Jilin	Hebei
2009	Inner Mongolia	Shanxi	Jilin	Heilongjiang	Hebei
2010	Inner Mongolia	Heilongjiang	Shanxi	Jilin	Xinjiang
2011	Inner Mongolia	Shanxi	Heilongjiang	Jilin	Ningxia
2012	Inner Mongolia	Heilongjiang	Shanxi	Xinjiang	Jilin
2013	Inner Mongolia	Shanxi	Heilongjiang	Xinjiang	Ningxia
2014	Inner Mongolia	Xinjiang	Ningxia	Shanxi	Heilongjiang
2015	Ningxia	Xinjiang	Inner Mongolia	Shanxi	Heilongjiang
2016	Xinjiang	Ningxia	Inner Mongolia	Heilongjiang	Shanxi
2017	Inner Mongolia	Xinjiang	Ningxia	Heilongjiang	Shanxi
2018	Inner Mongolia	Ningxia	Xinjiang	Heilongjiang	Shanxi
2019	Inner Mongolia	Ningxia	Xinjiang	Heilongjiang	Shanxi

### Spatial autocorrelation and hotspot analysis

The incidence of human brucellosis in China from 2004 to 2019 showed positive spatial correlations (*p* < 0.05), which indicated an overall high-value clustering with a stable degree of clustering (Table 3).

**Table 3:** Global autocorrelation analysis of human brucellosis incidence in China from 2004 to 2019.

Year	Moran’s *I*	*z*-value	*p*-value
2004	0.1967	2.714	0.007
2005	0.1856	3.833	<0.001
2006	0.2011	3.688	<0.001
2007	0.2020	3.598	<0.001
2008	0.2467	4.300	<0.001
2009	0.2056	4.360	<0.001
2010	0.1900	4.340	<0.001
2011	0.1944	4.245	<0.001
2012	0.3532	4.661	<0.001
2013	0.4249	4.393	<0.001
2014	0.3251	3.260	0.001
2015	0.2593	2.716	0.007
2016	0.2298	2.459	0.014
2017	0.3022	3.118	0.002
2018	0.3471	3.925	<0.001
2019	0.2919	3.861	<0.001

The local spatial autocorrelation analysis suggested that there were three types of local clustering in terms of human brucellosis in China: High-High Cluster, Low-Low Cluster, and Low-High Outlier. In the northern region, Inner Mongolia was always the High-High Cluster, while in the southern region, Zhejiang, Jiangxi, Hunan, Guangdong and Guizhou provinces were always the Low-Low Cluster (S2 Fig).

The results of hotspot analysis (Getis-Ord Gi* statistics) showed that the hotspot areas (with statistical significance) were all distributed in the northern parts of China, including Inner Mongolia, Heilongjiang, Ningxia, Shanxi, Jilin, Liaoning, Shaanxi, Hebei and Gansu, with Inner Mongolia, Heilongjiang and Ningxia consistently in the hotspot areas from 2004 to 2019. In 2006–2009 and 2012–2013, a large number of significant hotspot areas (99% confidence) were found covering four provincial-level administrative units, Inner Mongolia, Heilongjiang, Jilin and Shanxi. In 2014–2019, Inner Mongolia, Ningxia and Gansu were hotspot areas (99% confidence). Hebei province was not statistically significant after 2013 and dropped out of the hotspot region; Gansu province has been a significant hotspot region since 2009 (S3 Fig).

### Spatial panel data analysis

Based on the results of the standardized regression coefficients, the inventory of cattle, beef yield and gross pastoral product in the Northeast, gross pastoral product in the Northern coast, the inventory of cattle, the inventory of sheep, beef yield and gross pastoral product in the middle reaches of the Yellow River, the inventory of cattle in the Northwest and Tibet, gross pastoral product in the Eastern coast, beef yield and gross pastoral product in the Southern coast, milk yield and wool yield in the middle reaches of the Yangtze River, the inventory of cattle and gross pastoral product in the Southwest were not significant at the significance level of 5%, so these variables were not applicable for the corresponding region (Table 4).

**Table 4:** Results of univariate analysis (standardized regression coefficients).

Geographical regions	Socioeconomic factors (importance of ranking of coefficients)							Meteorological factors								Season		
								Average air temperature				Average relative humidity						
	1^st^	2^nd^	3^rd^	4^th^	5^th^	6^th^	7^th^	Lag 0	Lag 1	Lag 2	Lag 3	Lag 0	Lag 1	Lag 2	Lag 3	S1	S2	S3
North																		
Northeast	Milk yield (1.139)*	Wool yield (0.518)*	Mutton yield (0.380)*	Inventory of sheep (0.128)*	Gross pastoral product (-0.076)	Inventory of cattle (0.013)	Beef yield (0.006)	0.772*	-0.686	-1.898*	-0.494*	-0.072	-0.030	2.212×10^−4^	-0.036	0.727*	0.397*	-0.275*
Northern coast	Milk yield (1.339)*	Beef yield (-1.265)*	Mutton yield (1.137)*	Inventory of cattle (-0.521)*	Wool yield (0.187)*	Inventory of sheep (0.151)*	Gross pastoral product (-0.073)	0.642*	0.918*	0.997*	0.908*	-0.273*	-0.261*	-0.207*	-0.122*	0.377*	0.235*	-0.118
the middle reaches of the Yellow River	Milk yield (0.616)*	Wool yield (-0.374)*	Mutton yield (0.268)*	Inventory of sheep (-0.101)	Beef yield (0.094)	Gross pastoral product (-0.014)	Inventory of cattle (0.002)	0.781*	0.240*	-0.377*	-0.866*	-0.202*	-0.213*	-0.174*	-0.091*	0.578*	0.442*	-0.118*
Northwest and Tibet	Wool yield (2.054)*	Milk yield (0.954)*	Beef yield (0.909)*	Mutton yield (0.838)*	Inventory of sheep (0.757)*	Gross pastoral product (0.276)*	Inventory of cattle (0.118)	0.599*	0.391*	0.098	-0.207*	-0.055	-0.021	0.053	0.137*	0.237*	0.309*	0.027
South																		
Eastern coast	Milk yield (-1.461)*	Wool yield (-0.720)*	Inventory of cattle (-0.520)*	Beef yield (-0.445)*	Mutton yield (-0.220)*	Inventory of sheep (0.135)*	Gross pastoral product (-0.076)	2.867*	2.394*	1.295*	0.518	-0.082	-0.006	0.094	0.010	0.393*	0.087	-0.184
Southern coast	Milk yield (-0.782)*	Inventory of sheep (0.633)*	Mutton yield (0.538)*	Inventory of cattle (-0.507)*	Beef yield (-0.106)	Gross pastoral product (0.074)	-	0.004	-0.041	-0.098	-0.205*	0.278*	0.231*	0.132*	0.111*	0.275*	0.235*	-0.033
the middle reaches of the Yangtze River	Mutton yield (0.982)*	Gross pastoral product (0.247)*	Inventory of sheep (0.169)*	Beef yield (0.128)*	Inventory of cattle (0.126*)	Milk yield (0.066)	Wool yield (0.019)	-0.907*	-0.833*	-1.013*	-1.065*	0.158*	0.147*	0.141*	0.136*	0.215*	0.173*	-0.015
Southwest	Milk yield (1.714)*	Mutton yield (1.044)*	Beef yield (0.836)*	Wool yield (-0.725)*	Inventory of sheep (0.189)*	Inventory of cattle (0.087)	Gross pastoral product (-0.029)	-0.189*	-0.133	-0.002	0.121	0.069*	0.037	0.009	-0.015	0.197*	0.148*	-0.006

Note: * indicates *p* < 0.05.

Our study analyzed the ranking of the variables’ contribution of the eight geographical regions. The variation of standardized regression coefficients for the inventory of cattle ranged from -0.521 to 0.126, ranged from -0.128 to 0.757 for the inventory of sheep, ranged from -1.265 to 0.909 for beef yield, ranged from -0.220 to 1.137 for mutton yield, ranged from -0.725 to 2.054 for wool yield, ranged from -1.461 to 1.714 for milk yield, ranged from 0.247 to 0.276 for gross pastoral product, ranged from -0.907 to 2.867 for average air temperature (the same month), ranged from -0.833 to 2.394 for average air temperature (1-month lag), ranged from -1.898 to 1.295 for average air temperature (2-month lag), ranged from -1.065 to 0.908 for average air temperature (3-month lag), ranged from -0.273 to 0.278 for average relative humidity (the same month), ranged from -0.261 to 0.231 for average relative humidity (1-month lag), ranged from -0.207 to 0.141 for average relative humidity (2-month lag), ranged from -0.122 to 0.137 for average relative humidity (3-month lag), ranged from 0.197 to 0.727 for S1, ranged from 0.148 to 0.442 for S2, and ranged from -0.275 to -0.118 for S3. (Table 4).

**Fig 4:**
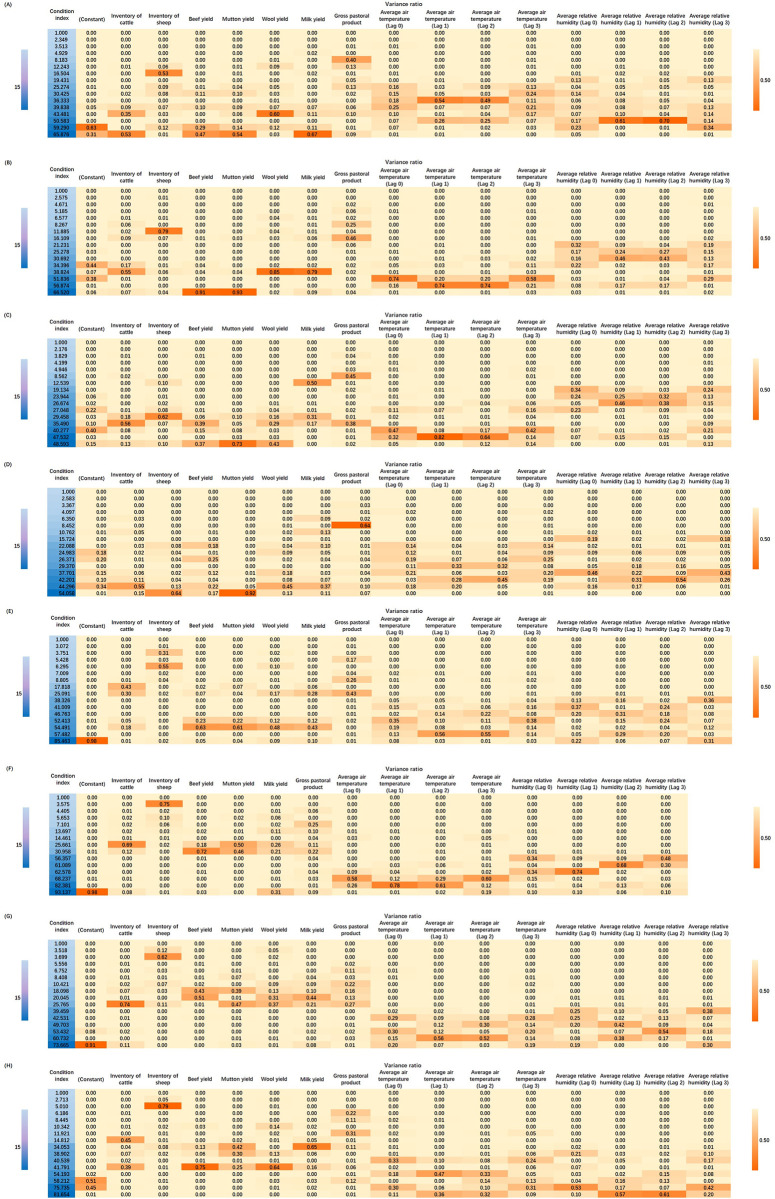
The results of the multi-collinearity test of socioeconomic and meteorological factors in eight geographical regions. Note: (A) Northeast, (B) Northern coast, (C) the middle reaches of the Yellow River, (D) Northwest and Tibet, (E) Eastern coast, (F) Southern coast, (G) the middle reaches of the Yangtze River, (H) Southwest.

The multi-collinearity test showed that milk yield, average air temperature (1-month lag) and average relative humidity (2-month lag) in the Northeast, the inventory of cattle, mutton yield, wool yield, average air temperature (1-month lag), average air temperature (2-month lag) and average relative humidity (1-month lag) in the Northern coast, average air temperature (1-month lag), average air temperature (2-month lag) and average relative humidity (2-month lag) in the middle reaches of the Yellow River, the inventory of cattle, the inventory of sheep, mutton yield, average air temperature (2-month lag), average air temperature (3-month lag), average relative humidity (the same month), average relative humidity (1-month lag) and average relative humidity (2-month lag) in the Northwest and Tibet, average air temperature (1-month lag) in the Eastern coast, beef yield, mutton yield, wool yield, average air temperature (1-month lag) and average air temperature (2-month lag) in the Southern coast, average air temperature (1-month lag) in the middle reaches of the Yangtze River, average air temperature (1-month lag), average air temperature (2-month lag), average relative humidity (1-month lag) and average relative humidity (2-month lag) in the Southwest were excluded (Fig 4). The final independent variables included in the spatial panel model were the inventory of cattle, the inventory of sheep, beef yield, mutton yield, wool yield, milk yield, gross pastoral product, the same month’s, two and three months lagged average air temperature, the same month’s, one and three month(s) lagged average relative humidity and seasonal effect (S1, S2, S3).

As for model applicability test, if LM-error was significant at a significance level of 5% and LM-lag was not, SEM should be used. If LM-lag was significant and LM-error was not, SLM should be adopted. If both LM-error and LM-lag were significant, robust LM tests were required. If both robust LM-error and robust LM-lag were significant, SDM should be run. If robust LM-error was significant and robust LM-lag was not, SEM should be used. If robust LM-lag was significant and robust LM-error was not, SLM should be adopted. Fixed effects model was chosen if Hausman test was significant at a significance level of 5%, and random effects model otherwise (Table 5).

**Table 5:** Spatial panel model selection.

Geographical regions	LM-error		LM-lag		Robust LM-error		Robust LM-lag		Hausman		Final model
	$\chi_{LM}^{2}\;(1)$	*p*-value	$\chi_{LM}^{2}\;(1)$	*p*-value	$\chi_{RLM}^{2}\;(1)$	*p*-value	$\chi_{RLM}^{2}\;(1)$	*p*-value	*χ*^2^(1)	*p*-value	
North											
Northeast	123.470	<0.001	94.364	0.001	64.350	<0.001	35.244	0.004	111.440	<0.001	SDM with fixed effect
Northern coast	145.770	<0.001	186.540	<0.001	13.498	<0.001	54.265	<0.001	6.901	0.907	SDM with random effect
the middle reaches of the Yellow River	15.035	<0.001	11.407	<0.001	176.420	<0.001	172.790	<0.001	2.668	0.999	SDM with random effect
Northwest and Tibet	2.253	0.133	15.032	<0.001	-	-	-	-	4.318	0.889	SLM with random effect
South											
Eastern coast	10.344	0.001	26.303	<0.001	15.863	<0.001	31.822	<0.001	75.144	<0.001	SDM with fixed effect
Southern coast	50.942	<0.001	64.389	<0.001	3.909	0.048	17.356	<0.001	45.598	<0.001	SDM with fixed effect
the middle reaches of the Yangtze River	387.950	<0.001	489.680	<0.001	1.092	0.296	102.820	<0.001	5.148	0.881	SLM with random effect
Southwest	113.490	<0.001	367.600	<0.001	89.936	<0.001	344.070	<0.001	31.137	0.019	SDM with fixed effect

Note: LM: Lagrange multiplier

As shown in Table 6, the inventory of cattle was negatively correlated with human brucellosis incidence in the Eastern coast, Southern coast and the middle reaches of the Yangtze River. The inventory of sheep was positively correlated with human brucellosis incidence in the Northeast, Northern coast and Eastern coast. Mutton yield was positively correlated with human brucellosis incidence in the middle reaches of the Yellow River, Eastern coast and the middle reaches of the Yangtze River, and negatively correlated with human brucellosis incidence in the Northeast. Beef yield was positively correlated in the Northwest and Tibet and the middle reaches of the Yangtze River, and negatively correlated in the Eastern coast. Milk yield was positively correlated with human brucellosis incidence in the Northern coast, the middle reaches of the Yellow River, Northwest and Tibet and Southwest, and negatively correlated in the Eastern coast and Southern coast. Wool yield was positively correlated with human brucellosis incidence in the Northeast and Northwest and Tibet, while the middle reaches of the Yellow River and Southwest was negatively correlated. Gross pastoral product was positively correlated with human brucellosis incidence in the middle reaches of the Yangtze River. The same month’s average air temperature was positively correlated with human brucellosis incidence in the Northwest and Tibet, Eastern coast and Southwest. Average air temperature with a lag period of two months was positively correlated with human brucellosis incidence in the Northeast. Average air temperature with a lag period of three months was negatively correlated with human brucellosis incidence in the middle reaches of the Yellow River and the middle reaches of the Yangtze River, and positively correlated in the Sothern coast. The same month’s average relative humidity was positively correlated with human brucellosis incidence in the Southern coast, the middle reaches of the Yangtze River and Southwest. As for seasonal effect, human brucellosis incidence in the Northeast, the middle reaches of the Yellow River and Eastern coast went up during spring. The incidence went up in the Northeast, Northern coast and the middle reaches of the Yellow River during summer, and also went up in the Northeast and the middle reaches of the Yellow River during autumn. The spatial autocorrelation coefficients of SDM and SLM were statistically significant, while no statistically significant correlation was found in the Northwest and Tibet. Among the above eight geographical regions, there was no statistically significant correlation between human brucellosis incidence and average air temperature with a lag period of one month and average relative humidity with a lag period of one to three months.

**Table 6:** Regression coefficients of spatial panel data analysis for eight geographical regions in China from 2004 to 2019.

Geographical regions	(Constant)	Wool yield	Milk yield	Beef yield	Mutton yield	Inventory of sheep	Inventory of cattle	Gross pastoral product	Average air temperature	Average relative humidity	Season			Spatial autoregressive coefficient
											S1	S2	S3	*ρ*
North														
Northeast	-1.322*	3.205×10^−5^*	-	-	-0.066*	6.696×10^−4^*	-	-	0.020* (Lag 2)	-	0.195*	0.333*	0.136*	0.382*
Northern coast	-3.259*	-	0.003*	0.003	-	0.001*	-	-	-0.032 (Lag 3)	0.006 (Lag 0)	0.126	0.254*	0.083	0.390*
the middle reaches of the Yellow River	-1.906*	-9.525×10^−6^*	0.001*	-	0.008*	-	-	-	-0.012* (Lag 3)	-0.001 (Lag 1)	0.085*	0.226*	0.108*	0.356*
Northwest and Tibet	-7.023*	4.043×10^−5^*	0.011*	0.175*	-	-	-	-4.364×10^−4^	0.018* (Lag 0)	-0.003 (Lag 3)	0.200	-0.008	-0.140	0.031
South														
Eastern coast	-0.087	1.387×10^−4^	-0.037*	-0.710*	0.183*	0.003*	-0.033*	-	0.131* (Lag 0)	-	0.294*	0.249	-0.102	0.150*
Southern coast	1.616	-	-0.146*	-	-	4.435×10^−4^	-0.003*	-	0.043* (Lag 3)	0.020* (Lag 0)	0.040	0.087	-0.029	0.244*
the middle reaches of the Yangtze River	-2.890*	-	-	0.025*	0.130*	-2.497×10^−4^	-0.002*	3.211×10^−4^*	-0.016* (Lag 3)	0.010* (Lag 0)	-0.048	0.045	0.012	0.549*
Southwest	-6.810*	-1.271×10^−4^*	0.034*	0.001	-0.002	2.380×10^−4^	-	-	0.026* (Lag 0)	0.010* (Lag 0)	0.060	-0.068	-0.159	0.286*

Note: * indicates *p* < 0.05. ‘-’ indicates not applicable for that region.

## Discussion

Brucellosis is one of the most common and neglected zoonotic infectious diseases worldwide [27], which has caused irreparable economic losses to the animal husbandry worldwide and also posed a serious threat to public health in most countries [28]. Brucellosis has been listed as one of the animal infectious diseases that urgently needs to be controlled and eradicated [29,30]. We analyzed the trends of human brucellosis incidence on temporal and spatial scales in China from 2004 to 2019, and examined the socioeconomic factors, meteorological indicators and season effect associated with human brucellosis incidence. The temporal distribution showed a clear seasonal trend in human brucellosis incidence, with cases concentrated between March and July and peaking in May, which was consistent with the breeding period of cattle and sheep [31].

The spatial distribution indicated that human brucellosis was mainly concentrated in the northern regions of China before 2011, and rapidly spread to the southern coast and southwestern regions from 2011 onwards [11]. The incidence of human brucellosis remained consistently high in Inner Mongolia, Shanxi and Heilongjiang. Spatial autocorrelation analysis revealed the spatial clustering effect of human brucellosis in China during the 16-year period, but the clustering areas varied over time. High-High Cluster areas were concentrated in the northern regions, mainly because pastoral areas were mainly located in the north of China, which was conducive to the development of animal husbandry and thus paved the way for human brucellosis. While areas such as Zhejiang and Guangdong province in the southern region belonged to Low-Low Cluster areas. The possible reason for this was that the southern regions had relatively less exposure to livestock and the incidence of human brucellosis is lower. Hotspot analysis suggested that the hotspot areas were all located in the north of China, especially in Inner Mongolia and adjacent provinces or autonomous regions, which was corresponding to the above-mentioned and previous findings [32].

By constructing the spatial panel model, we explored the driving factors of human brucellosis incidence in China. With respect to socioeconomic factors, human brucellosis incidence was negatively correlated with the inventory of cattle in the Eastern coast, Southern coast and the middle reaches of the Yangtze River. Northeast, Northern coast and Eastern coast showed a positive correlation between human brucellosis incidence and the inventory of sheep, which was consistent with the results of other studies in China [14,33–35]. Both cattle and sheep have the potential risk of transmitting *Brucella* to humans [30], and sheep in particular are the main risk factor for brucellosis [33], so the surveillance and control efforts by health authorities should be further strengthened. Human brucellosis incidence of was positively correlated with mutton yield in the middle reaches of the Yellow River, Eastern coast and the middle reaches of the Yangtze River, and negatively correlated in the Northeast. The incidence of human brucellosis was positively correlated with beef yield in the Northwest and Tibet and the middle reaches of the Yangtze River, and negatively correlated in the Eastern coast. Human brucellosis incidence was positively correlated with milk yield in the Northern coast, the middle reaches of the Yellow River, Northwest and Tibet and Southwest, and negatively correlated in the Eastern coast and Southern coast. Additionally, the incidence of human brucellosis was positively associated with wool yield in the Northeast, and Northwest and Tibet, and negatively correlated in the middle reaches of the Yellow River and Southwest. It has been pointed out that with the improvement of living standards, the demand for beef and mutton in the southern regions has greatly increased, resulting in the boosting circulation of cattle, sheep and their livestock products from the northern pastoral areas [36], which, together with inadequate protective measures, has greatly increased the risk of infection through exposure to *Brucella*-contaminated livestock and their products. What’s more, intensive animal husbandry could also bring the potential risk of human brucellosis [37,38]. Therefore, it is significant to enhance the quarantine of livestock, strengthen the controls on livestock products to prevent contaminated products from entering the market [39] and strengthen the sterilization of animal products in food processing. Human brucellosis incidence was positively correlated with gross pastoral product in the middle reaches of the Yangtze River, which was consistent with the findings of Zhao et al. [40]. The improvement of residential standards has accelerated the development of animal husbandry and the processing and transportation of livestock products, increasing the exposure of animal hosts [39]. In addition, high incidence of human brucellosis may be attributed to inadequate preventive measures and low awareness of brucellosis [11,41]. It is noteworthy that non-brucellosis endemic areas with low GDP and large numbers of cattle and sheep are also likely to become high incidence areas in the future [40], so it is recommended that the authorities take measures to strengthen risk prevention awareness.

The changes of human brucellosis risk are uncertain, with lagged effects influenced by several factors [12,13]. As for meteorological factors, human brucellosis incidence was positively associated with the same month’s average air temperature in the Northwest and Tibet, Eastern coast and the Southwest, positively associated with average air temperature at a lag time of 2-month in the Northeast, and positively associated with average air temperature at a lag time of 3-month in the Southern coast, which was similar to the findings of previous studies and explained the non-linear and lagged effect of meteorological factors on human brucellosis [42]. Appropriate temperatures provide a favorable environment for *Brucella* and also create good conditions for host animals to breed, thereby increasing the risk of infection for those exposed to these infected animals [13]. Consistent with the findings of Faramarzi *et al*. [43], human brucellosis incidence was positively correlated with the same month’s average relative humidity in the Southern coast, the middle reaches of the Yangtze River and the Southwest, which was supported by the findings of Yang *et al* [18, 44]. High-humidity environments can affect the enzymatic activity of *Brucella* and also its survival and transmission [12]. Therefore, health authorities should pay more attention to the impact of changes of relative humidity in the process of human brucellosis prevention and control.

Moreover, in spring, the incidence of human brucellosis was high in the Northeast, the middle reaches of the Yellow River and Eastern coast. During summer, human brucellosis incidence of was high in the Northeast, Northern coast, and the middle reaches of the Yellow River. In autumn, the incidence of human brucellosis was high in the Northeast and the middle reaches of the Yellow River. That revealed the seasonal effect of human brucellosis. The spatial autocorrelation coefficients of SDM and SLM were statistically significant except the Northwest and Tibet, indicating that human brucellosis incidence of one area was positively affected by the incidence of its neighbors. In other words, spatial clustering was observed, which was consistent with the above-mentioned results on spatial autocorrelation. Thus, the spatial interactions among neighbors were important and necessary in explaining variability in human brucellosis incidence. Joint prevention and control across multiple regions may reduce costs and increase efficiency.

There are several limitations in the present study. Firstly, as a class B notifiable infectious disease in China, human brucellosis must be reported online through the National Notifiable Diseases Surveillance System within 24 hours. However, under-diagnosis remains a serious problem, which could underestimate the incidence of human brucellosis. Secondly, individual-level information on the characteristics of the patients, the species of human brucellosis and the vaccination coverage of animals nationwide could not be obtained. Thirdly, given that the present study was based on the ecological study design and spatial panel model, and the latter focused on the observed population, which meant the results could not be used to make inferences about individuals, thus ecological fallacies were inevitable. In addition, spatial weight matrix may not fully reflect the complicated association among different areas. The above shortcomings are expected to be solved in the future studies.

## Conclusions

Overall, the human brucellosis epidemic area in China is expanding year by year, and there was significant spatial clustering. Inner Mongolia and its adjacent provinces or autonomous regions were hotspot areas of human brucellosis. The inventory of cattle and sheep, beef yield, mutton yield, wool yield, milk yield, gross pastoral product, average air temperature (the same month, 2-month lagged and 3-month lagged), average relative humidity (the same month) and season variability were found to play an important role in the development of human brucellosis. Therefore, it is high time that health authorities enhance the quarantine of livestock, strengthen the sterilization of animal products and implement health education for the target group. The present study strengthens the understanding of the relationship between socioeconomic, meteorological factors and the spatial heterogeneity of human brucellosis in China, through which ‘One Health’-based strategies and countermeasures can be provided for the government to tackle the brucellosis menace.

## Supporting information

S1 Table Descriptions of yearly meteorological indexes of eight regions in China from 2004 to 2019. (DOCX)

S1 Fig Spatial distribution of human brucellosis incidence in China from 2004 to 2019. Base layers were downloaded from Standard Map Service System by China Cartographic Publishing House (http://bzdt.ch.mnr.gov.cn/). No. GS(2020)4619. Note: the map of this study does not represent the true borders of administrative regions of China.
(TIF)

S2 Fig LISA (Local indicators of spatial association) clustering of the local Moran’s *I* for human brucellosis in China from 2004 to 2019. Base layers were downloaded from Standard Map Service System by China Cartographic Publishing House (http://bzdt.ch.mnr.gov.cn/). No. GS(2020)4619. Note: the map of this study does not represent the true borders of administrative regions of China.
(TIF)

S3 Fig Hot spot analysis for human brucellosis in China from 2004 to 2019. Base layers were downloaded from Standard Map Service System by China Cartographic Publishing House (http://bzdt.ch.mnr.gov.cn/). No. GS(2020)4619. Note: the map of this study does not represent the true borders of administrative regions of China.
(TIF)
