# Supplementary material for: Socioeconomic, meteorological factors and spatiotemporal distribution of human brucellosis in China between 2004 and 2019—A study based on spatial panel model
Source: PLoS Negl Trop Dis. 2023 Nov 13;17(11):e0011765. doi: 10.1371/journal.pntd.0011765 (PMC10681303; doi:10.1371/journal.pntd.0011765)
Supplement: S1 Table — (DOCX) [file pntd.0011765.s001.docx]

**S1 Table. Descriptions of yearly meteorological indexes of eight regions in China from 2004 to 2019.**

| Geographical regions | | Average air temperature in the same month (℃) | Average relative humidity in the same month (%) |
| --- | --- | --- | --- |
| **North** | |  |  |
|  | **Northeast** |  |  |
|  | Liaoning | 8.7 (7.5, 9.6) | 64.3 (57.1, 69.3) |
|  | Jilin | 6.7 (5.3, 7.7) | 59.0 (56.5, 64.8) |
|  | Heilongjiang | 5.1 (4.3, 6.6) | 63.1 (57.3, 69.1) |
|  | **Northern coast** |  |  |
|  | Beijing | 13.5 (12.9, 14.2) | 50.1 (47.0, 55.3) |
|  | Tianjin | 13.2 (12.6, 14.4) | 56.8 (52.5, 60.9) |
|  | Hebei | 14.3 (12.7, 15.1) | 55.9 (51.9, 64.5) |
|  | Shandong | 15.0 (14.2, 15.8) | 54.0 (51.8, 61.3) |
|  | **the middle reaches of the Yellow River** |  |  |
|  | Shanxi | 11.4 (10.6, 12.1) | 53.7 (49.8, 57.9) |
|  | Inner Mongolia | 7.8 (7.2, 9.0) | 46.3 (41.6, 50.6) |
|  | Henan | 15.9 (14.9, 16.9) | 58.8 (52.0, 62.3) |
|  | Shannxi | 14.6 (12.5, 19.4) | 60.0 (35.8, 67.8) |
|  | **Northwest and Tibet** |  |  |
|  | Tibet | 9.7 (9.3, 10.5) | 36.3 (30.1, 63.8) |
|  | Gansu | 8.2 (7.3, 8.7) | 55.9 (51.5, 59.4) |
|  | Qinghai | 6.5 (5.6, 7.0) | 56.5 (52.1, 58.3) |
|  | Ningxia | 10.8 (9.9, 11.5) | 48.6 (42.7, 52.3) |
|  | Xinjiang | 7.7 (6.7, 8.9) | 54.5 (48.2, 59.3) |
| **South** | |  |  |
|  | **Eastern coast** |  |  |
|  | Shanghai | 17.5 (16.9, 18.5) | 70.7 (67.3, 75.1) |
|  | Jiangsu | 16.7 (16.1, 17.4) | 71.3 (65.6, 73.1) |
|  | Zhejiang | 17.8 (17.2, 18.4) | 70.5 (67.7, 74.4) |
|  | **Southern coast** |  |  |
|  | Fujian | 20.7 (20.2, 21.3) | 71.8 (66.7, 76.7) |
|  | Guangdong | 22.4 (21.7, 23.2) | 74.5 (67.6, 80.3) |
|  | Hainan | 24.7 (23.4, 25.9) | 80.3 (77.9, 81.3) |
|  | **the middle reaches of the Yangtze River** |  |  |
|  | Anhui | 16.7 (16.2, 17.4) | 74.2 (68.4, 78.6) |
|  | Jiangxi | 18.8 (18.1, 19.3) | 71.6 (64.5, 76.3) |
|  | Hubei | 17.5 (16.5, 18.6) | 75.7 (66.9, 79.8) |
|  | Hunan | 18.1 (17.5, 19.4) | 72.8 (66.2, 82.1) |
|  | **Southwest** |  |  |
|  | Guangxi | 21.8 (20.9, 22.4) | 78.5 (73.3, 82.8) |
|  | Chongqing | 19.1 (18.4, 20.0) | 75.0 (69.3, 82.3) |
|  | Sichuan | 16.9 (16.1, 18.1) | 79.1 (72.5, 83.2) |
|  | Guizhou | 14.9 (13.8, 15.4) | 78.8 (73.4, 83.8) |
|  | Yunnan | 16.3 (15.4, 16.9) | 67.6 (62.3, 71.9) |
