# Supplementary figures and images for: Socioeconomic, meteorological factors and spatiotemporal distribution of human brucellosis in China between 2004 and 2019—A study based on spatial panel model
Source: PLoS Negl Trop Dis. 2023 Nov 13;17(11):e0011765. doi: 10.1371/journal.pntd.0011765 (PMC10681303; doi:10.1371/journal.pntd.0011765)

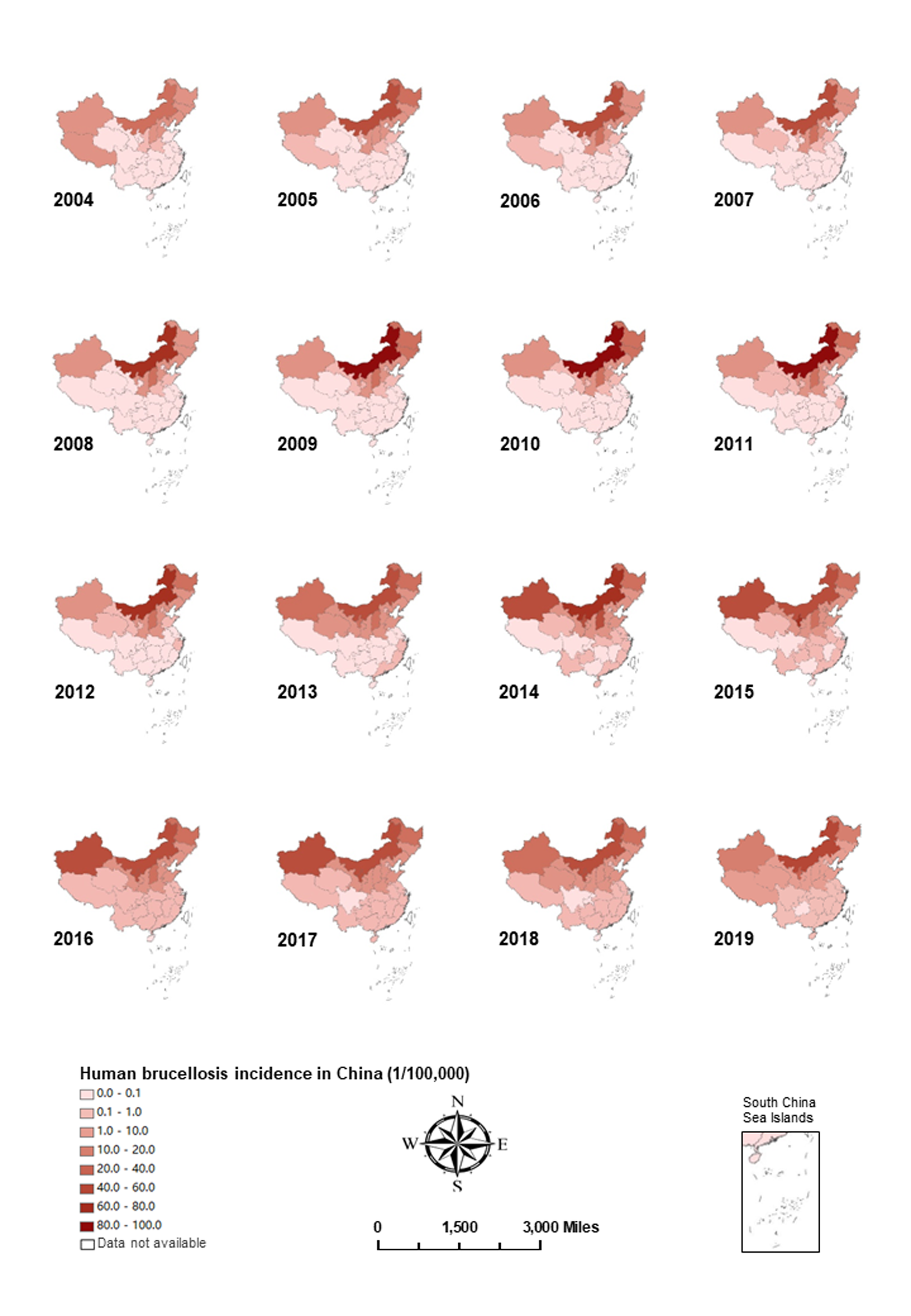

Supplement: S1 Fig — Base layers were downloaded from Standard Map Service System by China Cartographic Publishing House (http://bzdt.ch.mnr.gov.cn/). No. GS(2020)4619. Note: the map of this study does not represent the true borders of administrative regions of China. (TIF) [file pntd.0011765.s002.tif]

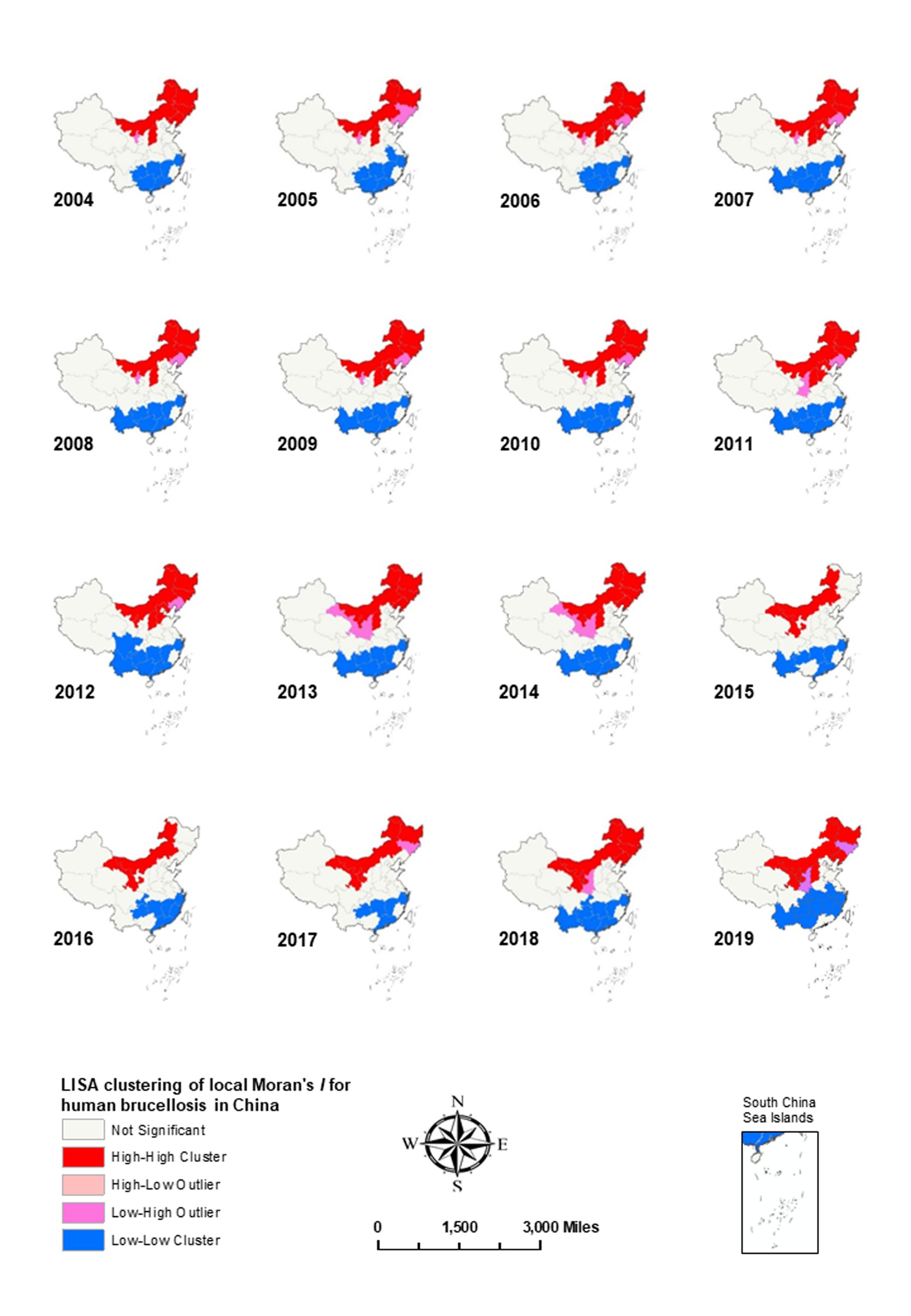

Supplement: S2 Fig — Base layers were downloaded from Standard Map Service System by China Cartographic Publishing House (http://bzdt.ch.mnr.gov.cn/). No. GS(2020)4619. Note: the map of this study does not represent the true borders of administrative regions of China. (TIF) [file pntd.0011765.s003.tif]

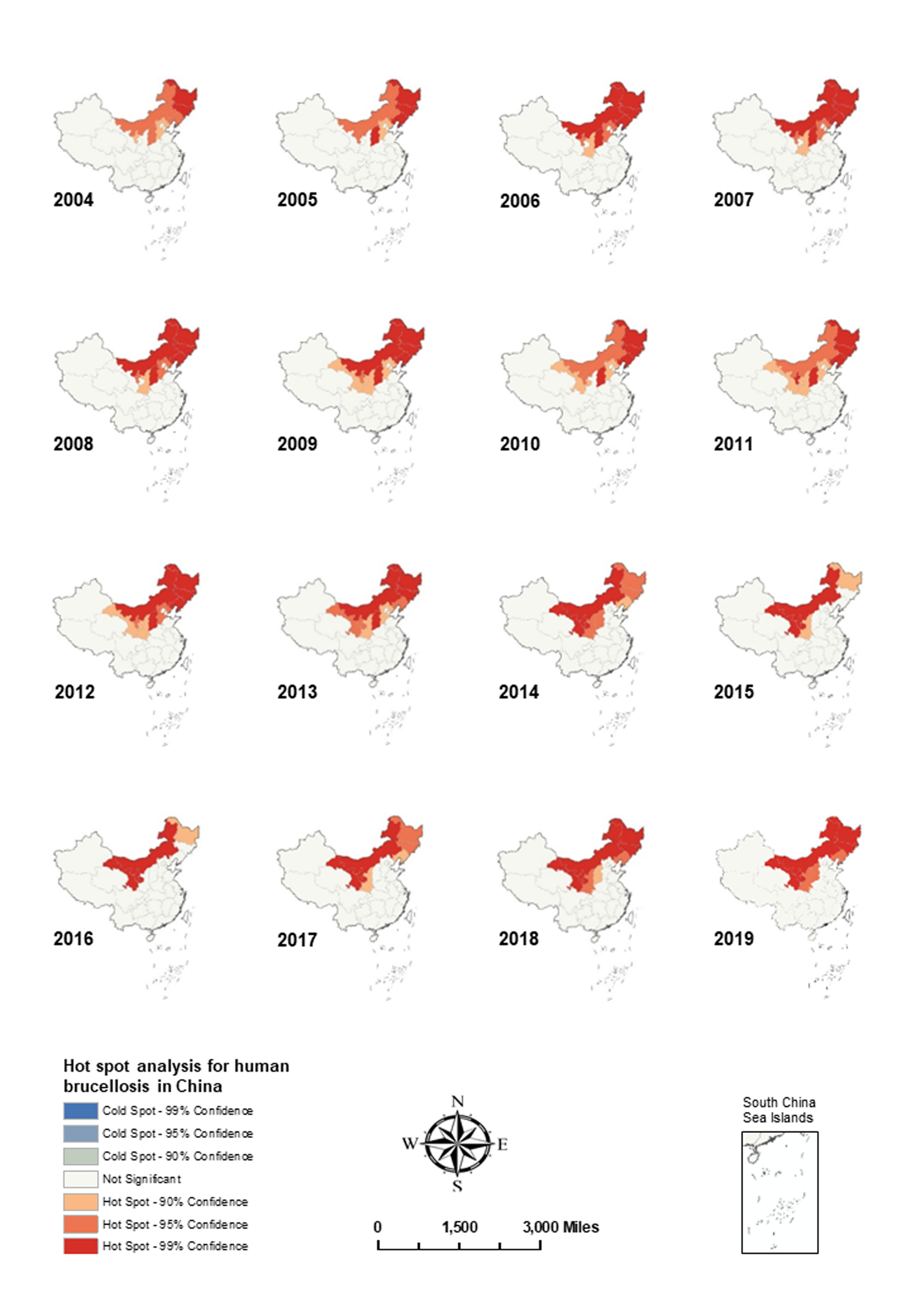

Supplement: S3 Fig — Base layers were downloaded from Standard Map Service System by China Cartographic Publishing House (http://bzdt.ch.mnr.gov.cn/). No. GS(2020)4619. Note: the map of this study does not represent the true borders of administrative regions of China. (TIF) [file pntd.0011765.s004.tif]
